# Growth Differentiation Factor 15 Is Induced by Hepatitis C Virus Infection and Regulates Hepatocellular Carcinoma-Related Genes

**DOI:** 10.1371/journal.pone.0019967

**Published:** 2011-05-23

**Authors:** Youhui Si, Xiuying Liu, Min Cheng, Maorong Wang, Qiaoling Gong, Yang Yang, Tianyi Wang, Wei Yang

**Affiliations:** 1 State Key Laboratory for Molecular Virology and Genetic Engineering, Institute of Pathogen Biology, Chinese Academy of Medical Sciences and Peking Union Medical College, Beijing, China; 2 Liver Disease Center of PLA, the 81st Hospital of PLA, Nanjing, China; 3 Department of Infectious Diseases and Microbiology, University of Pittsburgh, Pittsburgh, Pennsylvania, United States of America; Pohang University of Science and Technology, Republic of Korea

## Abstract

Liver fibrosis, cirrhosis, and hepatocellular carcinoma (HCC) are commonly induced by chronic hepatitis C virus (HCV) infection. We aimed to identify and characterize the involvement of previously screened cytokine GDF15 in HCV pathogenesis. We examined the GDF15 expression after HCV infection both *in vitro* and *in vivo*. Cultured JFH-1 HCV was used to determine the GDF15 function on virus propagation. GDF15 overexpression and RNA interference were employed to profile the GDF15-regulated genes, signaling pathways and cell biology phenotypes. The mRNA expression and protein secretion of GDF15 was dramatically increased in HCV-infected hepatoma cells, which maybe a host response to viral proteins or infection-induced cell stress. Patients infected with HCV had an average 15-fold higher blood GDF15 level than that of healthy volunteers. Three HCC individuals in the HCV cohort showed extremely high GDF15 concentrations. Transfection or exogenously supplied GDF15 enhanced HCV propagation, whereas knockdown of endogenous GDF15 resulted in inhibition of virus replication. Overexpressed GDF15 led to Akt activation and the phosphorylation of Akt downstream targeted GSK-3β and Raf. Several HCC-related molecules, such as E-cadherin, β-catenin, Cyclin A2/B1/D1, were up-regulated by GDF15 stimulation *in vitro*. Overexpression of GDF15 in hepatoma cells resulted in increased DNA synthesis, promoted cell proliferation, and importantly enhanced invasiveness of the cells. In conclusion, these results suggest that an elevated serum GDF15 level is a potential diagnostic marker for viral hepatitis, and GDF15 may contribute to HCV pathogenesis by altering the signaling and growth of host cells.

## Introduction

Hepatitis C virus (HCV) is an important human pathogen affecting an estimated 170 million people worldwide [1]. There is no HCV vaccine available, and the existing pegylated interferon/ribavirin standard-of-care antiviral therapy is only about 50% effective in genotype 1 HCV and poorly tolerated. Chronic HCV infection is often clinically asymptomatic but can frequently progress to liver fibrosis, cirrhosis and hepatocellular carcinomas (HCC). Although both viral proteins and dysregulated immunocytes have been implicated for causing liver diseases, the knowledge about HCV infection induced hepatopathogenesis is still limited. Therefore, non-invasive serum predictors of HCV infection and knowledge of their potential roles during disease progression are urgently needed to help adjust treatment regimens and understand pathogenic mechanisms.

Our previous work screened the possible involvement of growth differentiation factor 15 (GDF15) during HCV infection [2]. GDF15, also known as macrophage inhibitory cytokine 1, is a member of the transforming growth factor beta (TGF-β) cytokine superfamily. GDF15 was first cloned from human monocytoid cell line U937 and identified for inhibiting TNF-α production by macrophages [3]. As a circulating cytokine, the function of GDF15 may be systemic and complicated. Recent studies suggest that GDF15 might be involved in cell survival [4], cancer cell invasiveness [5], tumor-induced anorexia and weight loss [6]. Association studies suggest that GDF15 is dramatically induced in liver injury [7], cancers [8]–[10], cardiovascular diseases [11]–[13], thalassemia [14] and HPV-mediated cervical cancer [15]. However, an association between GDF15 and viral hepatitis has not been reported, so far.

In the present study, we identified the up-regulation of GDF15 in HCV-infected hepatoma cells and evaluated serum levels of GDF15 in cohorts of patients with chronic hepatitis C or hepatitis B. Additionally, we investigated the biological impact of increased GDF15 on hepatoma cells. We demonstrate that GDF15 is an HCV (HBV) induced host circulating biomarker and may play important roles during liver pathogenesis by regulating specific pathways and HCC-related genes.

## Results

### HCV induces GDF15 expression in cultured hepatoma cells

**Figure 1:**
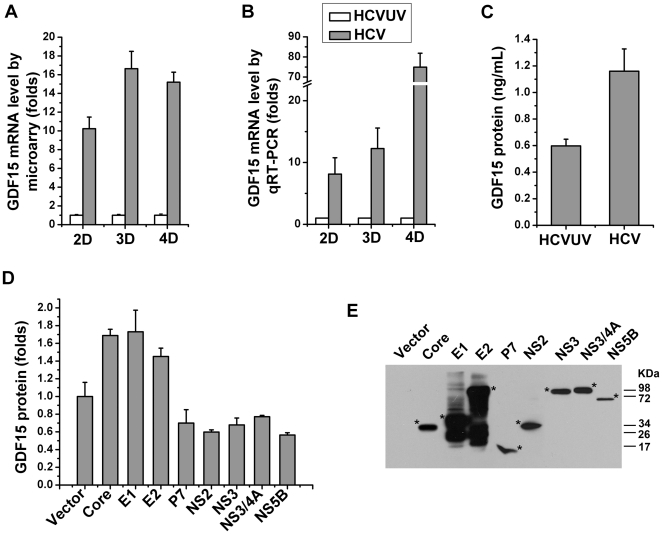
HCV infection up-regulates GDF15 expression *in vitro*. Huh7.5.1 cells were infected with JFH-1 HCVcc (HCV, MOI = 1) or the same amount of UV-inactivated virus (HCVUV) for 2 to 4 days (2–4 D). Total RNA was isolated for microarray and quantitative real-time RT-PCR analysis. (**A**) GDF15 mRNA expression abundances were extracted from microarray raw data[2] and represented as fold increases. (**B**) GDF15 mRNA levels were determined by real-time RT-PCR in the same manner as experiments designed in microarray analysis. (**C**) Concentrations of GDF15 protein in the medium supernatants sampled on cultured Huh7.5.1 cells 3 days post HCV or HCVUV infection. (**D**) Huh7.5.1 cells seeded in a 24-well plate were transfected with 1.5 µg of plasmid DNA, expressing the indicated viral proteins or an empty vector alone as a control. Three days after transfection, the levels of secreted GDF15 in culture supernatants were quantified by the ELISA method. (**E**) By immunobloting, the successful expression of above Flag-tagged individual viral proteins was confirmed. The position of each protein was indicated with a star sign.

Our previous study revealed several HCV regulated serum factors, in which GDF15 was constantly and dramatically induced by HCVcc infection [2]. With microarray analysis, GDF15 mRNA showed a 10-fold to 16-fold increase in HCVcc-infected Huh7.5.1 cells (Fig. 1A). Figure 1B showed the confirmation of GDF15 induction using qRT-PCR, which has a much broader dynamic and linear range compared with microarray. ELISA results showed that the supernatant concentration of GDF15 protein was 0.591±0.036 ng/mL in control cells and 1.175±0.103 ng/mL in HCV-infected cells (Fig. 1C). Furthermore, in order to elucidate which viral protein may be causing this upregulation, the selected individual HCV proteins were transiently transfected into Huh7.5.1 cells, and the secreted GDF15 protein was determined by ELISA. The results shown in Fig. 1D and 1E demonstrated that the expression of HCV structural proteins (Core, E1 or E2), but not non-structure proteins (P7, NS2, NS3, NS4A or NS5B), significantly induced GDF15 expression.

### GDF15 concentration in human blood is elevated in HCV- and HBV-infected patients

**Figure 2:**
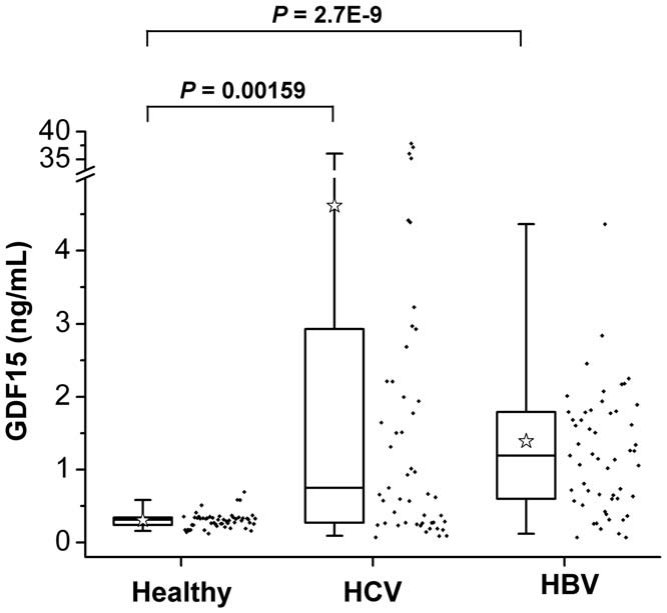
Blood GDF15 concentration is increased in HCV- or HBV-infected subjects. GDF15 concentrations in blood from healthy volunteers (n = 53) and patients with chronic hepatitis C (HCV, n = 54) or hepatitis B (HBV, n = 56) were analyzed by ELISA, and the results are depicted as box (25th percentile and 75th percentile) and whisker (5th and 95th percentiles) plots. The median was indicated as an open star. Data scatter plots are also demonstrated.

An increased plasma level of GDF15 has been documented in several human diseases [11], [14]. To understand the possible correlation between the GDF15 level and hepatitis C in a clinical setting, sera from healthy volunteers and treatment naïve HCV-infected patients were isolated for a GDF15 concentration measurement. Meanwhile, the HBV-infected cohort was also included as a control. Our major judgment for grouping was the presence of viral nucleic acids and antibodies. The basic characteristics of participants are summarized in Table 1. Determined with a commercial ELISA kit, the concentration of GDF15 in the healthy group ranged from 0.12 to 0.69 ng/mL, with a median of 0.30 ng/mL. However, the GDF15 level was significantly elevated in the HCV group (range 0.07–37.88 ng/mL, mean  = 4.62 ng/mL) and the HBV group (range 0.07–6.31 ng/mL, mean  = 1.39 ng/mL) (Fig. 2). The results from this *in vivo* study were consistent with those of the *in vitro* HCV-induced GDF15 up-regulation shown in Fig. 1. To further demonstrate the possible association of disease with the GDF15 level, more detailed characteristics about HCV participants are summarized in Table S1. HCV viral loads were not linearly correlated to the level of GDF15 expression. However, extremely high GDF15 levels were observed in three patients with HCC (13.83, 35.18 and 37.21 ng/mL respectively, indicated with an asterisk in Table S1).

**Table 1:** Basic Information about the Study Population.

	Healthy	HCV	HBV
**Total**	n = 53	n = 54	n = 56
**Age, years**	41 (21–69)	48 (20–70)	41 (18–76)
**Sex male, %**	n = 29, 54.7%	n = 30, 55.6%	n = 45, 80.4%

### GDF15 positively regulates HCV infection

**Figure 3:**
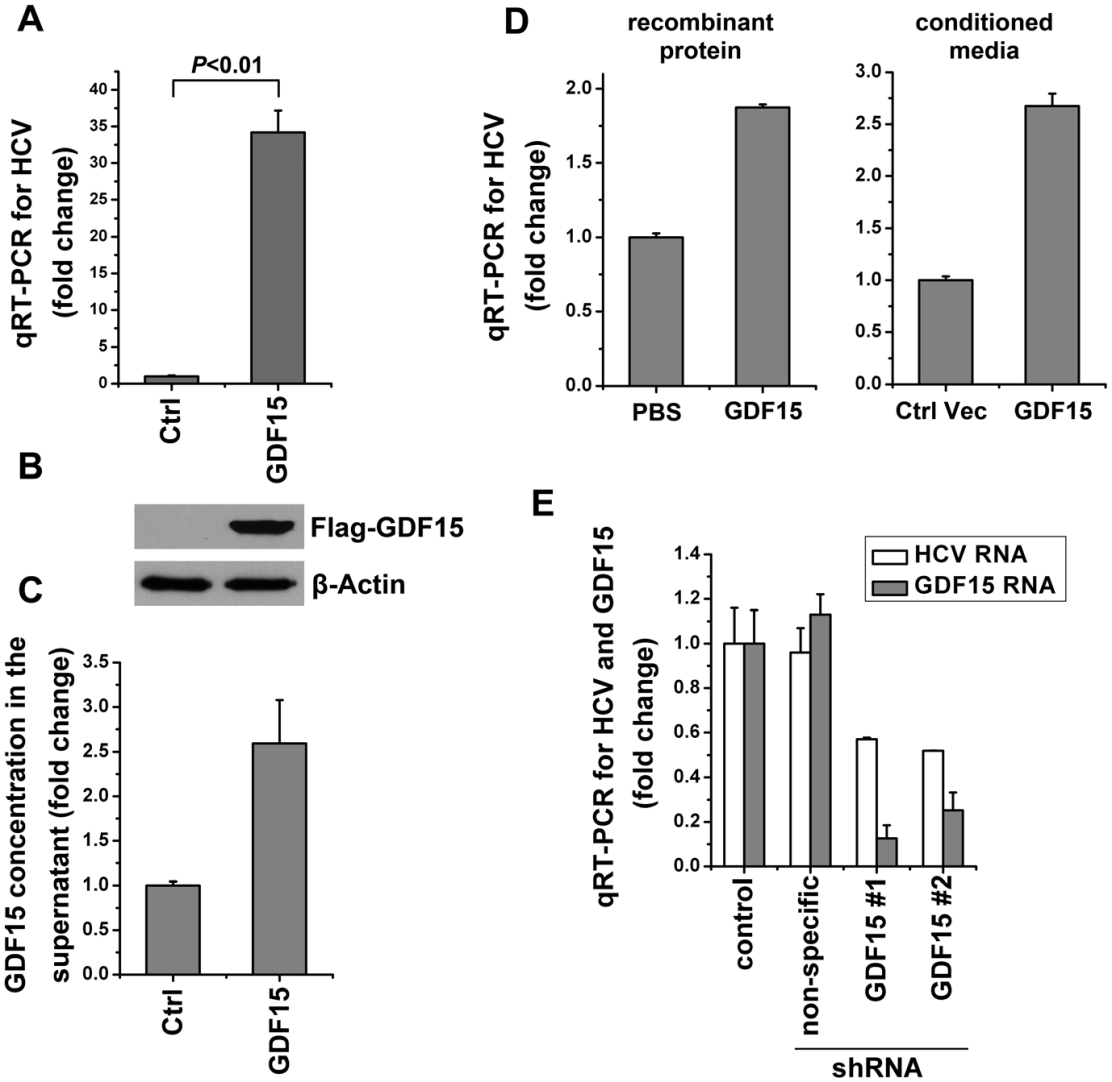
GDF15 positively regulates HCV infection. (**A**) Naïve Huh7.5.1 cells stably expressing human GDF15 or an empty vector as control (Ctrl) were cultured in 24-well plates and infected with JFH-1 HCVcc (MOI = 1) respectively. Forty eight hours post virus infection, total cellular RNA was isolated for HCV genomic RNA quantification using real-time RT-PCR. (**B**) The above cells were lysed for the detection of Flag-tagged GDF15 expression by immunobloting with the anti-Flag antibody (M2). Beta-Actin served as the sample equal loading control. (**C**) Besides the confirmation of the intracellular overexpression of transfected GDF15, the cell culture supernatants were collected from naïve Huh7.5.1 cells stably expressing human GDF15 or an empty vector for determination of the secreted GDF15 by ELISA. (**D**) Naïve Huh7.5.1 cells were treated with exogenous recombinant GDF15 protein (3 ng/mL) or conditioned media from GDF15 transfected Huh7.5.1 cell culture supernatant for 12 hours before JFH-1 HCVcc infection (MOI = 1). Forty eight hours post virus infection, total cellular RNA was isolated for HCV genomic RNA quantification using real-time RT-PCR. (**E**) Knockdown of GDF15 inhibits HCV replication. Naive Huh7.5.1 cells were transduced with shRNAi lentiviral particles of targeting GDF15 (GDF15#1 and #2), empty vector (control) and non-specific targeting, and then infected with JFH-1 HCVcc (MOI  = 1) for 2 days. GDF15 mRNA and HCV viral RNA was quantified by real-time RT-PCR and plotted as fold changes. All experiments were conduct in triplicate, and error bars represent standard deviations.

To determine how GDF15 affects HCV propagation in hepatocytes, gain-of-function and loss-of-function approaches were employed. We developed Huh7.5.1 cells that stably overexpress human GDF15 and an empty vector as control, using retroviral-mediated gene transfer technology. Compared with control Huh7.5.1 cells, the cells stably overexpressing GDF15 revealed an over 30-fold higher viral RNA copy number after JFH-1 HCVcc infection (Fig. 3A). The overexpression of intracellular GDF15 and secreted GDF15 in above stably transfected Huh7.5.1 cells were confirmed by immunoblotting and ELISA respectively (Fig. 3B and 3C). Similarly, the exogenous GDF15 from both purified recombinant protein and stably transfected Huh7.5.1 cell culture supernatant was able to increase HCVcc propagation (Fig. 3D). Furthermore, because no pharmacological or chemical GDF15 inhibitors were reported, the endogenous GDF15 expression was knocked down with shRNAi in HCVcc-infected Huh7.5.1 cells. Compared with the empty vector control and non-specific shRNAi, HCV replication was significantly suppressed after down-regulation of GDF15 expression (Fig. 3E). Together, these results demonstrated that GDF15, as an HCV-induced cytokine, could positively regulate JFH-1 HCV infection in cell culture system.

### Akt signaling pathway is regulated by GDF15 in hepatoma cells

**Figure 4:**
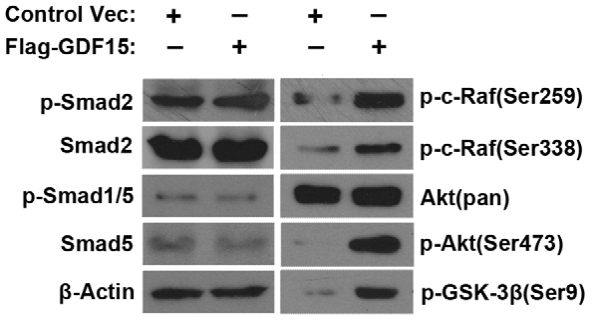
The Akt pathway is regulated by GDF15 overexpression in hepatoma cells. Huh7.5.1 cells stably expressing human GDF15 or an empty vector as control were analyzed for multiple signal cascades activation by immunobloting using the indicated antibodies.

To investigate the possible significance of HCV-induced GDF15 up-regulation on host response, several intracellular signaling pathways were measured in Huh7.5.1 cells stably overexpressing human GDF15. As a TGF-β superfamily member, GDF15 most likely uses the Smad pathway. However, the phosphorylation status of Smad1, 2 and 5 remained unchanged by GDF15 (Fig. 4). The total protein and phosphorylated-Smad3 levels were below the detection limitation in the cells we used (data not shown). The steady levels of inhibitory Smad (Smad7) and Co-Smad (Smad4) were not influenced by GDF15 (data not shown). However, Akt at Ser473 was significantly phophorylated by GDF15 (Fig. 4). Accordingly, two downstream targets of Akt, c-Raf (Ser259) and GSK-3β (Ser9), were also phosphorylated by GDF15 (Fig. 4). The activity of intracellular mitogen-activated protein kinases, including Erk1/2 and p38, was not altered by GDF15 in the current experimental system (data not shown). These results suggest that the Akt pathway might play a critical role in mediating GDF15-regulated physiological effects in hepatoma cells *in vitro*.

### Gene array analysis reveals the regulation of HCC-related genes by GDF15

**Figure 5:**
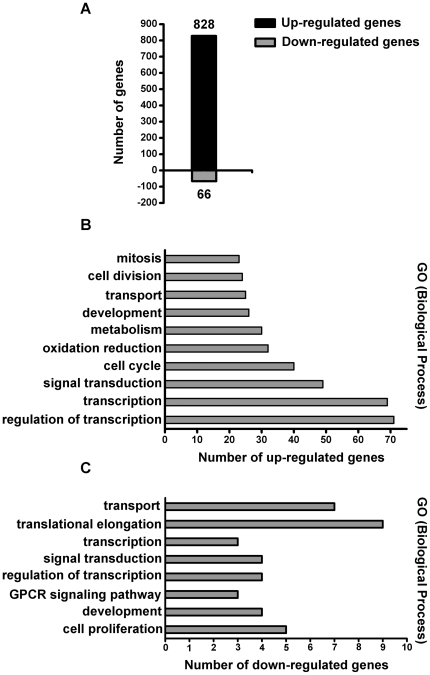
Profiling of GDF15 regulated gene expression. (**A**) Overall differentially expressed genes in GDF15-treated Huh7.5.1 cells. (**B**) and (**C**) demonstrate, respectively, the up-regulated and down-regulated genes that are grouped by the corresponding gene ontology biological process. Interestingly, the majority of genes up-regulated by GDF15 are involved in cellular signal transduction and transcription regulation.

To systematically profile the GDF15-regulated genes in hepatoma cells and to elucidate the possible role of GDF15 on biological processes, high-density oligonucleotide microarrays were hybridized with samples prepared from GDF15- or PBS-treated Huh7.5.1 cells. We found that a total of 894 genes were differentially expressed in response to GDF15 treatment based on the 2-fold cut-off range, and a majority (828 genes, about 92.6%) of the genes is up-regulated (Fig. 5A). According to the descriptions outlined by the Gene Ontology Biological Process, the differentially expressed genes were found to belong to a variety of functional categories (Fig. 5B, 5C). We assessed the expression of a set of genes that were previously reported by several groups with their potential involvement in HCC and summarized the results in Table 2. These HCC-associated genes include AFP, β-catenin, cyclin B1, c-myc, FOXO1A, IFNAR1, SOCS2, EGF, E-cadherin, CDKN2A, and IGFBP3 etc. Interestingly, more than half of the selected genes showed alterations in their expression. These results suggest that as an upstream regulator, excess GDF15 can sufficiently change the expression homeostasis of the HCC-associated gene setting.

**Table 2:** Expression level of HCC-associated genes in GDF15-treated Huh7.5.1 cells.

Genes	PBS treatment	GDF15 treatment	*p* Value	Fold (GDF/PBS)
PCNA	3436.5±341.5	18403.5±1221.2	0.00357	5.36
Cyclin A2	2035±140.0	7147±673.2	0.00892	3.51
IGFBP3	610±63.6	1937±233.3	0.01621	3.18
Cyclin B1	3188.5±767.2	9498.5±393.9	0.00921	2.98
CDK2	539.5±113.8	1527±113.1	0.01295	2.83
E-cadherin	1830.5±495.7	5000.5±1085.4	0.06411	2.73
β-catenin	5582.5±761.6	14596±189.5	0.00377	2.61
c-myc	3978.5±146.4	7181.5±608.8	0.01858	1.81
Cyclin D1	4533±233.3	7604.5±518.3	0.01670	1.68
SOCS2	59±5.7	71±7.1	0.20177	1.20
TGF-α	249±36.8	257±41.0	0.85627	1.03
AFP	36242±2907.6	36955.5±1689.3	0.79244	1.02
CDK4	10174.5±253.9	10303±264.5	0.66920	1.01
EGF	477±137.2	457.5±108.2	0.88907	0.96
FOXO1A	1079±364.9	1038±422.8	0.92679	0.96
IFNAR1	310.5±6.4	286±42.4	0.50409	0.92
CDKN2A	2896.5±600.3	2375±377.6	0.40759	0.82

### GDF15 modulates the cell cycle, proliferation and invasiveness of hepatoma cells

**Figure 6:**
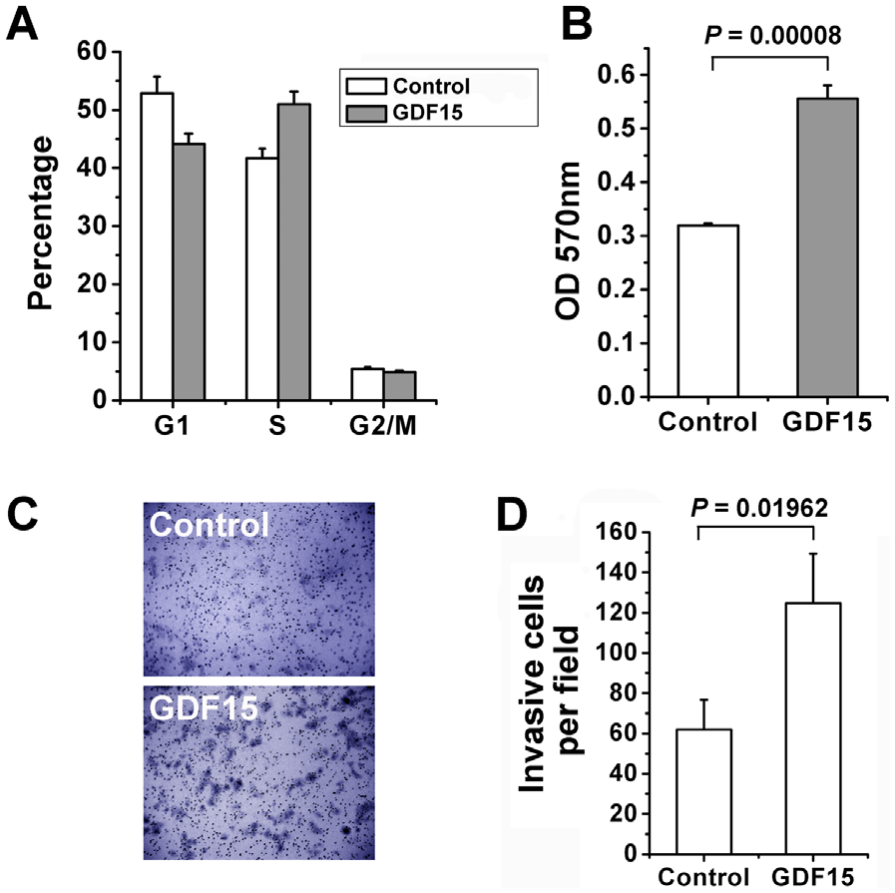
GDF15 regulates hepatoma cell cycle, proliferation and invasion. (**A**) Engineered Huh7.5.1 cells stably overexpressing GDF15 or an integrated empty vector control were harvested at exponential growth stage and stained by PI for cell cycle distribution analysis. (**B**) 10^4^ of above two types of cells were seeded in 96-well plate for cell proliferation assay with a MTT method. (**C**) The transwell invasion assay was performed as described in Materials and Methods using above GDF15 overexpressing and control cells. Magnification in the invasion assay is 200×. (**D**) The normalized ratio of the *in vitro* invasion assay.

To investigate the possible effect of GDF15 on cell biological characteristics, we engineered Huh7.5.1 cells that stably overexpress GDF15 or an empty vector as control. With flow cytometry analysis of DNA content, we observed that the percent of cells present in DNA synthesis phase of the cell cycle was increased by GDF15 overexpression (Fig. 6A). Meanwhile, GDF15 showed an enhancement of hepatoma cell proliferation (Fig. 6B). Using a similar experimental design, we further observed that overexpression of GDF15 promoted the invasiveness of Huh7.5.1 cells (Fig. 6C&D). These results suggest that GDF15 regulates multiple cell growth related phenotypes.

## Discussion

Different mechanisms have been proposed to explain HCV pathogenesis, including fibrogenesis and HCC. Among these mechanisms, an involvement of the virus-host interactions has been suggested by a number of *in vitro* and *in vivo* studies. The identification of HCV infection-regulated host serum factors allows us to explore predictive biomarkers, and to mechanistically investigate the host response upon infection. In this study, we have shown that the HCV infection is characterized by higher-than-normal GDF15 levels, and elevated GDF15 potentially regulates both HCV replication and host HCC-related signaling pathway and genes.

The TGF-β superfamily of ligands plays a pivotal role in the regulation of a wide variety of physiological processes from development to pathogenesis. GDF15 was originally discovered as a factor in immune regulation [3] and was subsequently considered a host responsive factor linked to tissue injury [7], cardiovascular events [11]–[13] and cancers [8], [9]. However, the correlations between the GDF15 expression level and virus infections (especially for hepatitis viruses) are undefined. We observed that 37 of 54 (68.5%) HCV and 50 of 56 (89.3%) HBV patients had a GDF15 level higher than the average GDF15 level in the healthy volunteers in the control group. Notably, the induction of GDF15 in both HCV and HBV infected patients suggests that GDF15 probably is a non-specific liver injury responsive cytokine in viral hepatitis and other hepatic diseases. As a circulating cytokine, GDF15 may function by transducing signals to target cells through the autocrine, paracrine and endocrine systems. So far, a variety of GDF15 biological functions has been reported, such as an anti-apoptotic in cardiomyocytes [16], metastasis in prostate cancer cells [17], motoneuron development [18], osteoclast differentiation [19], iron overloading and erythropoiesis [14], [20]. Therefore, it is possible that HCV infection might exert a systemic influence on extrahepatic manifestations during disease using GDF15 as a communicator.

Previous studies reported that the TGF-β1 level was elevated in chronic hepatitis C liver biopsy samples and urine [21], [22], but the association of the cytokine level and disease progression has not yet been clearly concluded. In the HCV-infected patients, we tried to assess the possible relationship between serum GDF15 levels and several disease characteristics, including viral load, genotypes and liver disease progression. Limited by the small number of subjects in the cohorts, we did not observe an accurate correlation, with the exception of 3 HCC patients that had extremely high levels of circulating GDF15 (Table S1, indicated with asterisk). This result suggests the possible involvement of GDF15 in HCV-related liver carcinogenesis and needs to be further verified in an extensive study with a large cohort. Several signal transduction cascades and molecules have been implicated to be involved in hepatocarcinogenesis [23], [24], although the detailed regulatory mechanisms remain to be elucidated. Our results demonstrated that Smad signaling, the classical intracellular cascade used by TGF-β superfamily members, was not activated by GDF15 in Huh7.5.1 cells. However, significant phosphorylation of Akt/GSK-3β was detected upon GDF15 overexpression (Fig. 4). Akt, a serine/threonine kinase, is responsible for regulation at multiple levels of cell growth and apoptotic processes, as well as liver carcinoma [25]. When the PI3K/Akt pathway is activated, GSK-3β, one of the major downstream substrates of Akt, is phosphorylated and then inhibited, which impairs the proteosome-dependent degradation of targets molecules, such as cyclin D1 and β-catenin [26], [27]. Also, unconventional activation of Akt was observed in HCC [28]. In addition, we identified a panel of important cancer-related genes, which were differentially expressed upon GDF15 stimulation. These genes included oncogenes β-catenin, E-cadherin, c-myc, cyclins, CDK2 and IGFBP3. A number of these genes were reported to be dysregulated in HCC. Importantly, we found that increased GDF15 regulated liver cell proliferation, cell cycle progression, even cell invasiveness. These results suggest that as an early and upstream response host factor of HCV infection, GDF15 might play profound roles in multiple cellular events.

This study also, for the first time, offers evidence that increased GDF15 expression promotes JFH-1 based HCVcc replication in cultured hepatoma cells. Moreover, knockdown of GDF15 by RNA interference suppresses HCV replication (Fig. 3). The biological role of TGF-β in HCV replication was controversially reported. A dose-dependent effect of TGF-β1 on the enhancement of HCV replication was observed [29], whereas Murata and colleagues reported the suppression of the HCV replicon by TGF-β via cell growth arrest [30]. Results from this study suggest that GDF15 up-regulation may be a host positive feedback mechanism for HCV infection. The broad effects of GDF15 on immune and transcription regulation might be involved in the modulation of HCV replication. Hopefully, approaches that specifically target GDF15 signaling will suppress HCV infection and the severity of liver disease.

Taken together, these findings provide evidence that host GDF15 is produced and secreted at high levels during HCV infection, which is probably caused by either viral agents or host stress/injury, or by both. We demonstrated that GDF15 may be normally required for efficient HCV infection and, as a homeostasis cytokine, may systematically regulate HCC-related signal transduction pathways and genes. Therefore, we propose that GDF15 represents a potential biomarker and interventional target for hepatitis C or B. A further large-scale association study is urgently needed for the predictive significance of GDF15 in viral hepatitis.

## Materials and Methods

### Cells and reagents

The human hepatoma cell line Huh7.5.1 was provided by Dr. Francis V. Chisari (The Scripps Research Institute, La Jolla, CA). HEK293T cells were obtained from ATCC. All cell lines were maintained in Dulbecco's Modified Eagle Medium (Invitrogen, Carlsbad, CA) supplemented with 1% penicillin and streptomycin (Gibco), 1% NEAA (Gibco), and 10% fetal bovine serum (Gibco) in a 37°C 5% CO_2_ atmosphere. Recombinant human GDF15 was obtained from R&D Systems (Minneapolis, MN). All antibodies were obtained from Cell Signaling (Danvers, MA).

### HCV cell culture viral infections and quantitative real time RT-PCR (qRT-PCR)

The production of JFH-1 (genotype 2a) HCV cell culture virus (HCVcc) was performed as previously described [2]. Inactivation of HCVcc by UV was achieved by exposing virus-containing supernatant in a Petri dish (0.5 cm depth) to a 254 nm UV source at 1.8 Jm-2 per second for 30 minutes. This inactivated virus was designated HCVUV. Infection of Huh7.5.1 cells, seeded in 24-well plates the day before, was performed in 500 µL of medium containing HCVcc (MOI = 0.5∼1). After the infection was allowed to proceed for 3 hours at 37°C, the unbound virus was washed away with phosphate-buffered saline (PBS), and the medium was replaced. At various time points (48, 72, or 96 hours) post infection, the cells or culture media were collected for the quantification of GDF15 or HCV. For the GDF15 treatment, the recombinant protein was added to Huh7.5.1 cells 24 hours prior to microarray analysis. Quantification of HCV RNA was performed by qRT-PCR, as previously described [31].

### Patients and healthy volunteers

Serum samples were drawn from hospital patients diagnosed with HCV or hepatitis B virus (HBV) infection. As a control, healthy volunteers with comparable age and gender distribution to the HCV- or HBV-infected patients were recruited from clinic physical examinations. At the time of enrollment, the written informed consent was obtained for the collection, storage of serum, and the purpose of the study. The experimental design and protocol were reviewed and approved by the ethics committee of Institute of Pathogen Biology. The patients' HCV or HBV status was confirmed by the presence of detectable serum HCV-RNA or HBV-DNA (Cobas Taqman test, Roche). Patients with a co-infection of HCV and HBV were excluded from the cohorts. Analysis of viral nucleic acids confirmed that healthy volunteers were free of HCV and HBV. There were 54 HCV-infected and 56 HBV-infected subjects, who were all treatment naïve. The subjects' serum and clinical data were extracted from medical records and laboratory reports.

### Measurement of GDF15 concentrations

The GDF15 protein levels in serum and cell culture supernatants were determined using a sandwich ELISA kit (R&D Systems), according to the manufacturer's instructions. The reaction was read under 450 nm with a SpectraMax M5 Multi-Mode Microplate Reader (Molecular Devices, Sunnyvale, CA). Each sample was analyzed in triplicate for statistical analysis. The GDF15 concentration of each sample was calculated using a standard curve and the measured absorbance.

### Plasmids construction, transfection and Immunoblotting

Plasmids encoding individual HCV proteins were constructed as previously described [32]. Huh7.5.1 cells seeded in a 24-well plate were transiently transfected with 1.5 µg of plasmid DNA plus 3 µL of TransMax transfection reagent (Giantagen, Beijing, China). Forty-eight hours post transfection, the cell culture supernatants were clarified by centrifugation for the GDF15 ELISA. Human GDF15 cDNA was cloned between the *BamH*I/*Xho*I sites of the pMIR-cFlag plasmid for expression and to generate an C-terminal Flag-tagged GDF15 fusion protein. To knockdown GDF15, two short hairpin RNA vectors and a non-specific control vector were constructed based on the pQSupR retroviral RNAi system, as previously described [33]. The target sequences of shRNA are as follows: GDF15#1 (5′-AAC CTG CAC AGC CAT GCC CGG-3′), GDF15#2 (5′-AAC TCA GGA CGG TGA ATG GCT-3′), and non-specific (5′-AAC GAC CGA TCC GCA CCG GCT-3′). For intracellular signal transduction analysis, the Huh7.5.1 cells stably transfected with empty vector or GDF15 expressing plasmid were lysed and the immunoblotting procedure was performed according to the previous reference[34] and antibody manufacture's instruction.

### Microarrays analysis

Huh7.5.1 cells were treated with recombinant GDF15 (10 ng/mL) or PBS for 24 hours. The microarray hybridization and analysis were performed by CapitalBio Corporation (Beijing, China) using the 22K Human Genome Array. Only genes that were either induced or reduced more than 2-fold in any of the samples were considered to be significantly up- or down-regulated by GDF15. All data is MIAME (Minimum Information About a Microarray Experiment) compliant and that the raw data has been deposited in GEO with the accession number of GSE25397.

### Cell cycle, proliferation and in vitro invasion assay

Huh7.5.1 cells, that stably overexpress GDF15 or an empty vector as control, were stained with Propidium Iodide (PI) and analyzed on a BD FACSCanto II flow cytometer for cell cycle distribution. For cell proliferation assay, about 10^4^ cells were seeded in each well of 96-well plates and analyzed with a non-radioactive/colorimetric method according to the manufacture's instructions (Promega). The *in vitro* invasion assay was performed using a Matrigel-coated membrane matrix (BD Bioscience) in the insert of a 24-well culture plate according to the manufacture's instructions. The assays were performed in three replicates and repeated three times.

### Statistical analysis

Two sample independent *t*-test statistics was employed to evaluate differences in GDF15 levels in healthy, HCV and HBV cohorts. Data are presented as a percentage, median (25^th^ to 75^th^ percentiles), or mean (SD), as indicated.

## Supporting Information

Table S1 Clinical Characteristics of the Hepatitis C Individuals in the Study.
(DOC)
