# Supplementary material for: Growth Differentiation Factor 15 Is Induced by Hepatitis C Virus Infection and Regulates Hepatocellular Carcinoma-Related Genes
Source: PLoS One. 2011 May 23;6(5):e19967. doi: 10.1371/journal.pone.0019967 (PMC3100307; doi:10.1371/journal.pone.0019967)
Supplement: Table S1 — Clinical Characteristics of the Hepatitis C Individuals in the Study. (DOC) [file pone.0019967.s001.doc]

**Supplementary data**

**Table S1. Clinical Characteristics of the Hepatitis C Individuals in the Study.**

| **Patient No.** | **Sex** | **Age (*y*)** | **Diagnosis** | **HCV antibody** | **HCV RNA (*copies/mL*)** | **GDF15(ng/mL)** |
| --- | --- | --- | --- | --- | --- | --- |
| 1 | F | 59 | CHC | Positive | 9.83E+04 | 1.0132 |
| 2 | F | 66 | HC | Positive | 1.23E+07 | 0.5687 |
| 3 | M | 51 | CHC | Positive | 8.66E+06 | 2.9710 |
| 4 | F | 54 | HC, LC | Positive | 1.13E+07 | 1.7742 |
| 5 | M | 51 | HC, LC | Positive | 1.14E+07 | 0.9273 |
| 6 | M | 69 | HC, LC | Positive | 2.69E+05 | 0.9681 |
| 7 | M | 41 | HC, DM | Positive | 3.07E+07 | 0.2710 |
| 8 | F | 50 | HC | Positive | 2.24E+06 | 0.2490 |
| 9 | M | 64 | HC | Positive | 5.46E+03 | 1.9959 |
| 10 | M | 38 | HC | Positive | 1.00E+07 | 1.9396 |
| 11 | F | 58 | HC, LC | Positive | 8.04E+06 | 1.5122 |
| 12 | M | 39 | HC | Positive | 2.59E+04 | 0.2296 |
| 13 | M | 41 | CHC | Positive | 8.91E+05 | 13.2085 |
| 14 | M | 56 | HC | Positive | 1.40E+07 | 4.3891 |
| 15 | M | 35 | CHC | Positive | 2.45E+06 | 0.5952 |
| 16 | F | 40 | CHC | Positive | 1.21E+03 | 0.2637 |
| 17 | F | 24 | HC | Positive | 1.69E+04 | 0.2341 |
| 18 | F | 41 | CHC | Positive | 6.22E+05 | 0.3731 |
| 19 | M | 53 | HC | Positive | 1.82E+06 | 1.5039 |
| 20 | M | 43 | HC | Positive | 5.75E+06 | 0.1436 |
| 21 | F | 64 | HC, LC | Positive | 2.04E+06 | 3.2241 |
| 22 | F | 52 | HC, LC | Positive | 1.68E+05 | 0.4141 |
| 23 | M | 61 | CHC | Positive | 7.29E+05 | 14.2308 |
| 24 | M | 46 | CHC | Positive | 4.94E+05 | 0.6651 |
| 25 | F | 45 | HC | Positive | 1.10E+06 | 2.2103 |
| **26*** | F | 70 | CHC, HCC | Positive | 5.32E+03 | 13.8253 |
| 27 | M | 33 | CHC | Positive | 1.09E+07 | 0.1884 |
| 28 | M | 46 | HC | Positive | 1.64E+06 | 1.3113 |
| 29 | M | 37 | HC | Positive | 4.56E+05 | 0.2692 |
| 30 | F | 55 | HC, LC | Positive | 1.04E+05 | 0.7483 |
| 31 | M | 33 | HC | Positive | 9.17E+04 | 0.2750 |
| 32 | F | 55 | HC | Positive | 3.75E+06 | 4.4157 |
| 33 | M | 54 | CHC | Positive | 2.31E+06 | 2.9294 |
| 34 | F | 29 | HC | Positive | 3.07E+05 | 2.6864 |
| 35 | M | 47 | CHC | Positive | 2.88E+04 | 2.2137 |
| 36 | M | 57 | CHC | Positive | 9.97E+05 | 0.6190 |
| 37 | F | 45 | HC | Positive | 4.99E+06 | 0.2637 |
| 38 | F | 54 | CHC | Positive | 1.33E+06 | 0.1890 |
| 39 | M | 23 | CHC | Positive | 3.89E+04 | 0.5739 |
| 40 | M | 52 | CHC | Positive | 3.38E+07 | 0.0875 |
| 41 | F | 48 | CHC | Positive | 1.64E+05 | 1.6456 |
| 42 | F | 58 | HC, LC | Positive | 6.62E+06 | 0.3692 |
| 43 | M | 54 | HC | Positive | 1.50E+05 | 0.6575 |
| 44 | F | 46 | CHC | Positive | 1.11E+06 | 0.1715 |
| 45 | F | 47 | CHC | Positive | 4.24E+06 | 0.2386 |
| 46 | M | 47 | HC | Positive | 4.03E+06 | 0.2861 |
| 47 | F | 47 | CHC | Positive | 1.69E+06 | 7.0975 |
| 48 | M | 38 | CHC | Positive | 2.02E+07 | 0.0696 |
| 49 | M | 46 | HC | Positive | 1.94E+05 | 0.0901 |
| 50 | F | 22 | HC | Positive | 2.71E+07 | 5.8664 |
| 51 | M | 66 | HC, LC | Positive | 9.00E+05 | 37.8781 |
| **52*** | M | 56 | HC, HCC | Positive | 4.34E+05 | 35.1831 |
| 53 | M | 20 | HC | Positive | 5.19E+06 | 36.0263 |
| **54*** | F | 65 | HC, LC, HCC | Positive | 2.55E+06 | 37.2053 |

**HC: Hepatitis C; CHC: Chronic Hepatitis C (>6 months since diagnosis); LC: Liver Cirrhosis; HCC: Hepatocellular Carcinoma; DM: Diabetes Mellitus**
